# Associations of Healthy Lifestyle With All‐Cause and Cardiovascular Disease Mortality in Individuals With Cardiovascular‐Kidney‐Metabolic Syndrome: Two Prospective Cohort Studies

**DOI:** 10.1002/clc.70436

**Published:** 2026-08-05

**Authors:** Qiqi You, Wan Fu, Mi Xu, Yucen Wu, Aaron Mitchell Lett, Xiaoying Li, Menglin Fan, Shaoyong Xu

**Affiliations:** ^1^ Department of Endocrinology, Xiangyang Central Hospital Affiliated Hospital of Hubei University of Arts and Science Xiangyang China; ^2^ Center for Clinical Evidence‐Based and Translational Medicine Xiangyang Central Hospital, Affiliated Hospital of Hubei University of Arts and Science Xiangyang China; ^3^ Department of Metabolism, Digestion and Reproduction Imperial College London London UK; ^4^ College of Medicine Hubei University of Arts and Science Xiangyang Hubei China

**Keywords:** cardiovascular‐kidney‐metabolic syndrome, healthy lifestyle, mortality, NHANES, UK Biobank

## Abstract

**Background:**

Cardiovascular‐kidney‐metabolic (CKM) syndrome poses a major health risk. This study assessed the impact of a healthy lifestyle on all‐cause and cardiovascular disease (CVD) mortality in CKM individuals.

**Methods:**

We analyzed 306 831 and 9823 CKM individuals from UK Biobank and NHANES (2007–2018). A healthy lifestyle score was created based on seven factors: no current smoking, moderate drinking, healthy diet, regular physical activity, adequate sleep, low sedentary behavior and appropriate social connection. Primary outcomes were all‐cause and CVD mortality from linked health records and death registries.

**Results:**

Cox regression models showed that higher lifestyle scores were associated with reduced risks of all‐cause (hazard ratio [HR]: 0.81, 95% confidence interval [CI]: 0.80–0.82) and CVD mortality (HR: 0.82, 95% CI: 0.80–0.84) in UK Biobank. In NHANES, higher scores were associated with 15% (95% CI: 0.80–0.90) and 11% (95% CI: 0.81–0.99) lower mortality risks. Both regular physical activity (HRs: 0.85 and 0.89 for UK Biobank, 0.76 and 0.73 for NHANES) and low sedentary behavior (HRs: 0.85 and 0.91 for UK Biobank, 0.79 and 0.67 for NHANES) were related to reduced mortality risks (*p* < 0.05). Participants with non‐advanced CKM syndrome and a favorable lifestyle had the lowest mortality risks compared to those with advanced CKM syndrome and an unfavorable lifestyle.

**Conclusion:**

A healthy lifestyle, particularly regular physical activity and low sedentary behavior, was significantly associated with lower all‐cause and CVD mortality in individuals with CKM syndrome. These associations were stronger in the early stages, highlighting the importance of timely and targeted lifestyle interventions.

## Graphical abstract

In two large cohorts, a healthy lifestyle was associated with significantly lower all‐cause and cardiovascular mortality in individuals with cardiovascular‐kidney‐metabolic syndrome, compared with an unfavorable lifestyle. The protective associations were stronger in the early stages of cardiovascular‐kidney‐metabolic syndrome.

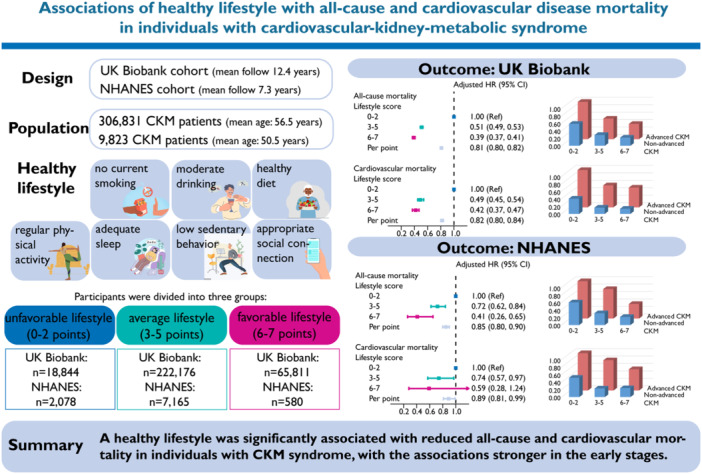

**Abbreviations**
AHA: American Heart Association
CI: confidence interval
CKD: chronic kidney disease
CKM: cardiovascular‐kidney‐metabolic
CVD: cardiovascular disease
HR: hazard ratio
NHANES: National Health and Nutrition Examination Survey
PIR: poverty income ratio

## 1 Introduction

The interconnections among obesity, type 2 diabetes, cardiovascular diseases (CVD), and chronic kidney disease (CKD) reflect a well‐established epidemiological pattern [1]. This clustering of multisystem dysregulation was recently formalized as cardiovascular‐kidney‐metabolic (CKM) syndrome by the American Heart Association (AHA) [2]. This syndrome imposed a substantial population burden: 89.5% of US adults (2011–2020) exhibited at least stage 1 of CKM syndrome, with 5.4% and 9.2% progressing to high‐risk stages 3‐4 [3]. The synergistic multi‐organ dysfunction in advanced CKM stages translated to alarming mortality risks, manifested by 158% and 273% excess mortality in stage 3 and 4 respectively, compared to stage 0 [4]. This escalating risk trajectory underscores the urgent need for targeted interventions.

Pharmacotherapies such as metformin, sodium‐glucose cotransporter 2 inhibitors, and glucagon‐like peptide‐1 receptor agonists have demonstrated cardio‐renal protective effects in populations with components of CKM syndrome, including prediabetes, type 2 diabetes, and CKD [5, 6, 7]. Robust evidence also confirms that healthy lifestyles are associated with reduced mortality risks in individual conditions such as type 2 diabetes, CVD, and CKD [8, 9], offering complementary advantages such as broad accessibility and low cost. However, the impact of lifestyle factors within the framework of CKM syndrome remains insufficiently characterized. It has been pointed out that education and support for healthy lifestyles may improve CKM health [10] and reduce the risk of cardio‐renal‐metabolic multimorbidity [11], yet critical evidence gaps persist. First, the stage‐dependent association between lifestyle adherence and mortality risk remains unquantified, particularly for advanced stages 3–4. Additionally, it remains unclear whether the associations between individual lifestyle components and mortality risks are heterogeneous. These gaps impede the development of personalized lifestyle recommendations for CKM populations.

Therefore, we used data from the UK Biobank and the National Health and Nutrition Examination Survey (NHANES) to evaluate the complex associations of the overall healthy lifestyle and its components with the risk of all‐cause and CVD mortality across different stages of CKM syndrome.

## 2 Materials and Methods

### 2.1 Study Design

Two independent databases were analyzed in this study, and the reporting conformed to the Strengthening the Reporting of Observational Studies in Epidemiology (STROBE) guidelines. One was the UK Biobank, a large population‐based cohort with previously described details [12]. The other was NHANES, a nationally representative survey carried out by the Centers for Disease Control and Prevention. While NHANES has disseminated cross‐sectional survey, examination, and laboratory data up to 2023, its public‐use mortality linkage was last updated to December 31, 2019 [13].

### 2.2 Study Population

From an initial pool of 502 392 participants in the UK Biobank, 125 024 were excluded for incomplete CKM syndrome data. Additional exclusions due to missing cause of death (*n* = 128), lifestyle scores (*n* = 66 279) and covariates (*n* = 4130) reduced the sample to 306 831. In the NHANES dataset (2007–2018, 59 842 participants), individuals were excluded if they were under 20 years (*n* = 25 072), lacked CKM syndrome data (*n* = 15 082), lacked death cause (*n* = 37), lacked lifestyle scores (*n* = 9005), or lacked covariates (*n* = 823). The remaining sample for analysis was 9823 (Supporting Information: eFigure 1).

### 2.3 Definition of CKM Syndrome

According to the AHA Statement, CKM syndrome were categorized into five stages [2, 14]. We defined four hierarchical components: (A) excess or dysfunctional adiposity (overweight/obesity, abdominal obesity, or dysfunctional adipose tissue); (B) metabolic risk factors; (C) CKD; and (D) CVD, with D1 (subclinical CVD) and D2 (clinical CVD). The very high‐risk Kidney Disease Improving Global Outcomes CKD stage and a high 10‐year predicted CVD risk were used as risk equivalents for D1 [3]. Stages were then defined as: Stage 0: no components present; Stage 1: component A only (without B, C, or D); Stage 2: component B or C (with or without A), but no D; Stage 3: D1 accompanied by at least one of A, B, or C; Stage 4: D2 accompanied by at least one of A, B, or C. Additional details were available in the eMethods.

### 2.4 Assessment of Lifestyle Factors

A seven‐item healthy lifestyle score was developed, covering four traditional factors (smoking, physical activity, alcohol consumption, diet) and three emerging ones (sleep duration, sedentary behavior, social connection) [9]. Each factor was dichotomized as low‐risk (1 point) versus others (0 point), with higher total scores (range 0–7) indicating greater adherence. Based on the sum, participants were categorized into unfavorable (0–2 points), average (3–5 points), and favorable (6–7 points) lifestyle groups. The thresholds for assigning 1 point were as follows.

Smoking: being a non‐current smoker [9, 15]. Physical activity: accumulating ≥ 150 min/week of moderate activity or ≥ 75 min/week of vigorous activity (or an equivalent mix). Drinking: moderate and regular intake (≤ 1 drink/day for females, ≤ 2 drinks/day for males); irregular drinkers (including never or occasional drinking) did not qualify. Diet: differed by database—in UK Biobank, consuming at least half of the 10 recommended food groups [9]; in NHANES, being in the top two quintiles of the Healthy Eating Index [16]. Sleep duration: 7–8 h/day, the mortality nadir in prospective cohorts [9, 17]. Sedentary behavior: < 4 h/day; TV‐watching served as a proxy for leisure sedentary behavior in UK Biobank [18], while NHANES used direct self‐report. Social connection: risk was defined as high in UK Biobank when participants met ≥ 2 of three conditions (living alone, social visits less than once monthly, and no participation in social activities at least weekly). In NHANES, living alone indicated high risk due to the unavailability of data on the other two dimensions.

### 2.5 Assessment of Other Factors

Demographic and health‑related information, including age, sex, ethnicity, education level, history of cancer, and medication use (for cholesterol, blood pressure or diabetes), was collected through self‑administered questionnaires. In UK Biobank, the Townsend deprivation index was employed to assess material deprivation, with higher scores indicating greater levels of deprivation. Medication use in UK Biobank was ascertained by the question “Do you regularly take any of the following medications,” with response options covering blood pressure medication, cholesterol lowering medication and insulin. We additionally identified diabetes medication using the treatment/medication codes from Data‐Field 20003. In NHANES, the poverty income ratio (PIR) was used as an indicator of household economic status, where lower PIR values reflect poorer financial conditions. Regarding medication use in NHANES, we extracted information from the following questionnaire items: current insulin use (DIQ050), use of diabetic pills (DIQ070), current use of prescribed medicine for hypertension (BPQ050A), and current use of prescribed medicine to lower blood cholesterol (BPQ100D).

Physical examination information included height, weight, waist circumference, systolic blood pressure, and diastolic blood pressure. Body mass index was calculated as weight (kg)/height (m^2^). Laboratory measurements covered blood glucose, glycated hemoglobin, triglycerides, high‐density lipoprotein cholesterol, total cholesterol, serum creatinine, urine creatinine, and urine microalbumin. The glomerular filtration rate was calculated using the 2021 CKD‐EPI creatinine equation, and the urinary albumin creatinine ratio was calculated as urine albumin/urine creatinine. In the UK Biobank, urine albumin measurements were unavailable for 349 525 of the 502 392 baseline participants. Consequently, CKD was defined solely based on glomerular filtration rate and International Classification of Diseases, 10th revision (ICD‐10) codes diagnoses. This approach prevented substantial sample loss but may have led to an underestimation of true CKD prevalence.

### 2.6 Ascertainment of Deaths

The primary outcomes included all‐cause and CVD mortality. The observation period ran from the baseline date to either the date of death or the administrative censoring date (UK Biobank: November 30, 2021, NHANES: December 31, 2019), whichever came first. For UK Biobank participants, death dates and causes were obtained through linked health records using ICD‐10 codes. For NHANES, mortality information was derived from a linkage to the National Death Index database, which directly provided death status, cause of death, and time‐to‐event data.

### 2.7 Statistical Analysis

Analysis was performed using Stata 17.0 and RStudio (2024.04.2), with *p* < 0.05 as significant. Baseline characteristics were described by healthy lifestyle scores, with continuous variables as mean ± standard deviation/median (interquartile range) and categorical variables as numbers (percentages).

Cox regression models were used to assess associations between healthy lifestyle scores and all‐cause/CVD mortality. The proportional hazards assumption was tested and met (all *p* > 0.05). Two adjustment schemes were used: Model 0 (unadjusted) and Model 1 (adjusted for age, sex, ethnicity, education, Townsend deprivation index in UK biobank, PIR in NHANES, body mass index, history of cancer, and CKM stage). We also estimated the change in mortality risk per one‐point increment in the lifestyle score. Beyond the overall score, the association of individual lifestyle factor with mortality was examined separately in patients with CKM syndrome.

In this study, CKM stage 3 and stage 4 were defined as advanced CKM, otherwise defined as non‐advanced CKM. Analyses were then performed separately within the non‐advanced and advanced subgroups, and combined effects of CKM stage (non‐advanced/advanced) and lifestyle score on mortality were explored. We further examined the lifestyle score as binary variables using three comparisons (3–7 vs. 0–2 points, 6–7 vs. 0–5 points, and 6–7 vs. 0–2 points). These comparisons were evaluated for all‑cause and CVD mortality separately in non‑advanced CKM, advanced CKM, and the full CKM population. Cohort‐specific hazard ratios (HRs) were pooled using inverse‑variance weighted meta‑analysis. A fixed‐effect model was applied as the default approach. When substantial heterogeneity was indicated by an *I^2^* statistic of 50% or higher, a random‑effects model was used instead.

Subgroup analyses explored potential effect modification by age (≥ 65 vs. < 65) and sex. Sensitivity analyses comprised the following approaches: excluding events within 2 years of baseline to minimize reverse causality; multiple imputation by chained equations for handling missing covariate data [19]; excluding baseline cancer to reduce potential confounding by pre‐existing poor health; constructing weighted lifestyle scores considering the effect size (*ln HR*) of each healthy lifestyle factor on mortality; adding body mass index (low risk: 18.5–25 kg/m^2^) to the lifestyle score; and adjusting for medication use (for cholesterol, blood pressure or diabetes).

## 3 Results

### 3.1 Baseline Characteristics

Among 306 831 UK Biobank (mean age: 56.5 years; 47.9% males) and 9823 NHANES (mean age: 50.5 years; 45.7% males) participants, 7.4% and 9.4% had CKM stage 4. Across all CKM stages, the majority of participants adhered to an average lifestyle pattern, and favorable lifestyles was more prevalent in the early stages (Supporting Information: eFigure 2). In both cohorts, higher lifestyle scores were associated with younger age, male sex, higher education levels, lower levels of substance deprivation, lower prevalence of comorbidities, and reduced medication use (all *p* < 0.05). Levels of body mass index, waist circumference, blood pressure, glycated hemoglobin and triglyceride were significantly lower in healthy lifestyle group than in unhealthy lifestyle group (all *p* < 0.001) (Table 1).

**TABLE 1:** Baseline characteristics of the study population.

Baseline characteristics^a^	Lifestyle score group (UK Biobank, *n* = 306 831)				Lifestyle score group (NHANES 2007–2018, *n* = 9823)			
	0–2 (*n* = 18 844)	3–5 (*n* = 222 176)	6–7 (n = 65,811)	*P* value	0–2 (*n* = 2078)	3–5 (*n* = 7165)	6–7 (*n* = 580)	*P* value
Age (year)	56.8 ± 7.9	56.5 ± 8.0	56.3 ± 8.2	< 0.001	54.2 ± 17.9	49.5 ± 17.9	48.6 ± 16.5	0.029
Males, n (%)	8765 (46.5)	104 181 (46.9)	34 090 (51.8)	< 0.001	893 (43.0)	3314 (46.3)	283 (48.8)	0.009
White, n (%) or Mexican American, n (%)	17 566 (93.2)	211 013 (95.0)	63 757 (96.9)	< 0.001	304 (14.6)	1066 (14.9)	61 (10.5)	< 0.001
College/University degree, n (%)	3425 (18.2)	74 040 (33.3)	29 223 (44.4)	< 0.001	376 (18.1)	2304 (32.2)	317 (54.7)	< 0.001
Townsend deprivation index	−0.4 (−2.8, 2.8)	−2.3 (−3.7, 0.2)	−2.6 (−3.9, −0.6)	< 0.001	—	—	—	—
Poverty income ratio	—	—	—	—	2.0 (1.1, 3.8)	2.7 (1.3, 4.9)	4.1 (2.0, 5.0)	< 0.001
Healthy lifestyle, n (%)								
No current smoking	10 021 (53.2)	200 969 (90.5)	65 349 (99.3)	< 0.001	947 (45.6)	5517 (77.0)	555 (95.7)	< 0.001
Moderate drinking	1810 (9.6)	92 694 (41.7)	56 843 (86.4)	< 0.001	283 (13.6)	2660 (37.1)	479 (82.6)	< 0.001
Healthy diet	554 (2.9)	28 548 (12.9)	31 527 (47.9)	< 0.001	295 (14.2)	3571 (49.8)	538 (92.8)	< 0.001
Regular physical activity	2507 (13.3)	115 123 (51.8)	61 165 (92.9)	< 0.001	182 (8.8)	2960 (41.3)	527 (90.9)	< 0.001
Adequate sleep	4023 (21.4)	144 613 (65.1)	61 973 (94.2)	< 0.001	385 (18.5)	4199 (58.6)	542 (93.5)	< 0.001
Low sedentary behavior	3991 (21.2)	154 293 (69.5)	63 256 (96.1)	< 0.001	136 (6.5)	2002 (27.9)	325 (56.0)	< 0.001
Appropriate social connection	10 411 (55.3)	203 580 (91.6)	65 195 (99.1)	< 0.001	1434 (69.0)	6469 (90.3)	573 (98.8)	< 0.001
History of cancer, n (%)	1645 (8.7)	16 914 (7.6)	4581 (7.0)	< 0.001	243 (11.7)	756 (10.6)	44 (7.6)	0.017
History of diabetes, n (%)	2026 (10.8)	12 264 (5.5)	2563 (3.9)	< 0.001	524 (25.2)	1282 (17.9)	63 (10.9)	< 0.001
History of hypertension, n (%)	11 468 (60.9)	121 484 (54.7)	32 103 (48.8)	< 0.001	1061 (51.1)	2740 (38.2)	173 (29.8)	< 0.001
History of chronic kidney disease, n (%)	1684 (8.9)	12 411 (5.6)	2705 (4.1)	< 0.001	523 (25.2)	1067 (14.9)	60 (10.3)	< 0.001
History of cardiovascular disease, n (%)	2540 (13.5)	17 208 (7.8)	4070 (6.2)	< 0.001	305 (14.7)	605 (8.4)	29 (5.0)	< 0.001
Diabetes medication, n (%)	1288 (6.8)	7460 (3.4)	1554 (2.4)	< 0.001	370 (17.8)	833 (11.6)	36 (6.2)	< 0.001
Cholesterol lowering medication, n (%)	4799 (25.5)	38 753 (17.4)	8949 (13.6)	< 0.001	629 (30.3)	1692 (23.6)	114 (19.7)	< 0.001
Blood pressure medication, n (%)	5377 (28.5)	46 250 (20.8)	10 609 (16.1)	< 0.001	852 (41.0)	2081 (29.0)	125 (21.6)	< 0.001
Body mass index (kg/m^2^)	28.9 ± 5.8	27.5 ± 4.6	26.1 ± 3.9	< 0.001	31.0 ± 7.6	29.1 ± 6.5	26.7 ± 5.0	< 0.001
Waist circumference (cm)	94.8 ± 14.9	90.6 ± 13.3	87.3 ± 12.1	< 0.001	104.4 ± 17.1	99.0 ± 15.9	93.3 ± 13.5	< 0.001
Systolic blood pressure (mmHg)	138.4 ± 18.7	138.0 ± 18.6	136.8 ± 18.4	< 0.001	126.3 ± 18.4	122.7 ± 17.6	121.0 ± 17.2	0.022
Glycated hemoglobin (mmol/mol)	36.3 (33.7, 39.5)	35.1 (32.7, 37.8)	34.7 (32.4, 37.2)	< 0.001	37.7 (34.4, 42.1)	36.6 (33.3, 41.0)	35.5 (33.3, 38.8)	< 0.001
Triglyceride (mmol/l)	1.7 (1.2, 2.5)	1.5 (1.1, 2.2)	1.4 (1.0, 1.9)	< 0.001	1.4 (1.0, 2.2)	1.3 (0.9, 2.0)	1.1 (0.8, 1.7)	< 0.001
High density lipoprotein cholesterol (mmol/l)	1.3 (1.1, 1.6)	1.4 (1.2, 1.7)	1.4 (1.2, 1.7)	< 0.001	1.3 (1.1, 1.6)	1.3 (1.1, 1.6)	1.4 (1.2, 1.7)	< 0.001
Cholesterol (mmol/l)	5.7 ± 1.2	5.7 ± 1.1	5.7 ± 1.1	< 0.001	4.9 ± 1.1	4.9 ± 1.0	4.9 ± 1.0	0.301
Serum creatinine (umol/l)	69.8 (60.7, 80.1)	70.7 (61.6, 81.2)	71.6 (62.4, 81.8)	< 0.001	75.1 (63.6, 90.2)	74.3 (62.8, 88.4)	76.0 (63.6, 88.0)	0.017
Glomerular filtration rate (ml/min per 1.73 m^2^)	94.1 ± 14.6	94.1 ± 12.9	94.7 ± 12.1	< 0.001	88.6 ± 24.8	94.1 ± 21.8	94.9 ± 18.3	< 0.001
Urinary albumin creatinine ratio (mg/g)	—	—	—	—	8.0 (5.0, 18.1)	6.7 (4.4, 12.2)	6.3 (4.2, 10.3)	< 0.001

^a^ Continuous variables were expressed as means ± standard deviations or medians (interquartile ranges), and categorical variables were expressed as numbers (percentages).

### 3.2 Associations Between Lifestyle Scores and Mortality in Individuals With CKM Syndrome

**FIGURE 1:**
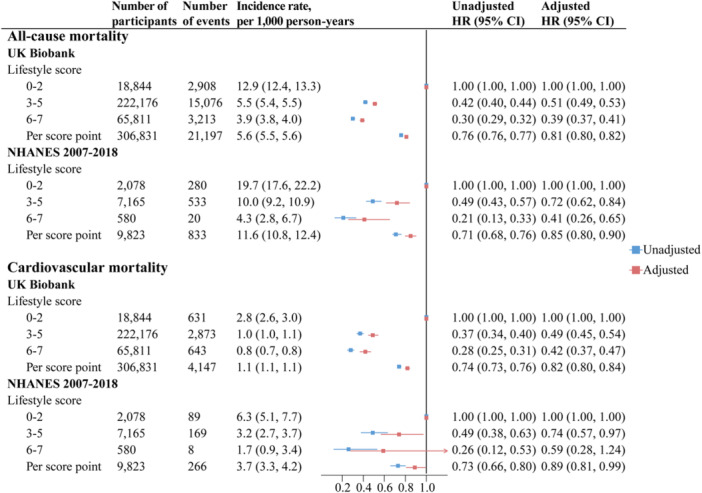
Association between lifestyle score and the risk of mortality in cardiovascular‐kidney‐metabolic syndrome. Footnote: Adjusted HR (95% CI) was calculated by adjusting age, sex, ethnicity, education, Townsend deprivation index (UK biobank), poverty income ratio (NHANES), body mass index, history of cancer, and cardiovascular‐kidney‐metabolic stage. CI, confidence interval; HR, hazard ratio.

Compared to participants at CKM stage 0, those at CKM stage 4 had the highest risk of all‐cause and CVD mortality in both cohorts (*p* < 0.05; Supporting Information: eTable 1). During a mean follow‐up of 12.4 years, UK Biobank recorded 21 197 all‐cause and 4147 CVD deaths. Each unit increase in lifestyle score was associated with significantly lower risks of all‐cause (HR: 0.81, 95% confidence interval [CI]: 0.80, 0.82) and CVD mortality (HR: 0.82, 95% CI: 0.80, 0.84). Participants with favorable lifestyles demonstrated substantially lower risks versus unfavorable lifestyle groups for all‐cause (HR: 0.39, 95% CI: 0.37, 0.41) and CVD mortality (HR: 0.42, 95% CI: 0.37, 0.47) (Figure 1).

In NHANES (mean follow‐up 7.3 years; 833 all‐cause and 266 CVD deaths), higher lifestyle scores were also associated with decreased risks of all‐cause (HR: 0.85, 95% CI: 0.80, 0.90) and CVD mortality (HR: 0.89, 95% CI: 0.81, 0.99). The favorable lifestyle group showed significantly lower all‐cause mortality (HR: 0.41, 95% CI: 0.26, 0.65) and non‐significantly lower CVD mortality (HR: 0.59, 95% CI: 0.28, 1.24) versus the unfavorable group (Figure 1).

Table 2 presented the association between individual lifestyle factors and mortality risks. In both cohorts, moderate drinking, regular physical activity, and low sedentary behavior were associated with lower risks of all‐cause mortality (all *p* < 0.05). Furthermore, regular physical activity and low sedentary behavior demonstrated significant inverse associations with CVD mortality (all *p* < 0.05).

**TABLE 2:** Association between individual lifestyle factor and the risk of mortality in cardiovascular‐kidney‐metabolic syndrome.

	All‐cause mortality		Cardiovascular mortality	
	Model 0	Model 1	Model 0	Model 1
UK Biobank				
No current smoking	0.49 (0.47, 0.51)***	0.50 (0.48, 0.52)***	0.44 (0.40, 0.47)***	0.46 (0.42, 0.50)***
Moderate drinking	0.82 (0.79, 0.84)***	0.85 (0.82, 0.87)***	0.85 (0.80, 0.90)***	0.86 (0.81, 0.92)***
Healthy diet	1.05 (1.02, 1.09)**	0.99 (0.95, 1.02)	1.12 (1.04, 1.21)**	1.06 (0.98, 1.14)
Regular physical activity	0.80 (0.78, 0.82)***	0.85 (0.83, 0.88)***	0.79 (0.74, 0.84)***	0.89 (0.84, 0.95)***
Adequate sleep	0.75 (0.73, 0.77)***	0.87 (0.85, 0.90)***	0.70 (0.66, 0.74)***	0.86 (0.80, 0.91)***
Low sedentary behavior	0.54 (0.52, 0.55)***	0.85 (0.82, 0.87)***	0.52 (0.49, 0.55)***	0.91 (0.86, 0.98)**
Appropriate social connection	0.58 (0.55, 0.60)***	0.72 (0.70, 0.75)***	0.51 (0.47, 0.56)***	0.67 (0.61, 0.73)***
NHANES 2007–2018				
No current smoking	0.45 (0.39, 0.51)***	0.88 (0.76, 1.02)	0.52 (0.41, 0.66)***	1.11 (0.86, 1.45)
Moderate drinking	1.19 (1.03, 1.36)*	0.82 (0.70, 0.95)*	1.30 (1.02, 1.66)*	0.89 (0.68, 1.17)
Healthy diet	1.03 (0.90, 1.18)	0.87 (0.75, 1.00)*	1.20 (0.94, 1.53)	1.02 (0.80, 1.31)
Regular physical activity	0.48 (0.41, 0.56)***	0.76 (0.64, 0.91)**	0.42 (0.31, 0.57)***	0.73 (0.53, 1.00)*
Adequate sleep	0.89 (0.78, 1.02)	0.91 (0.79, 1.05)	0.91 (0.71, 1.16)	0.94 (0.74, 1.21)
Low sedentary behavior	0.68 (0.58, 0.81)***	0.79 (0.67, 0.94)**	0.58 (0.42, 0.79)***	0.67 (0.48, 0.92)*
Appropriate social connection	0.42 (0.36, 0.49)***	0.89 (0.76, 1.05)	0.38 (0.29, 0.49)***	0.82 (0.62, 1.09)

*Note:* Model 0: unadjusted; Model 1: adjusted for age, sex, ethnicity, education, Townsend deprivation index (UK biobank), poverty income ratio (NHANES), smoking, drinking, diet, physical activity, sleep, sedentary behavior, social connection, body mass index, history of cancer, and cardiovascular‐kidney‐metabolic stage. *p < 0.05; **p < 0.01; ***p < 0.001.

### 3.3 Association Between Lifestyle Scores and Mortality in Non‐Advanced/Advanced CKM Stages

Within the non‐advanced CKM group in UK Biobank, individuals with favorable lifestyle had the lowest risk of all‐cause mortality (HR: 0.38, 95% CI: 0.36, 0.40) and CVD mortality (HR: 0.35, 95% CI: 0.30, 0.41). Similar inverse associations were observed in the advanced CKM group. In NHANES, a favorable lifestyle was associated with the lowest risk of all‐cause mortality in both non‐advanced (HR: 0.36, 95% CI: 0.18, 0.71) and advanced CKM stages (HR: 0.39, 95% CI: 0.21, 0.75). An average lifestyle was related to a 57% (HR: 0.43, 95% CI: 0.23, 0.79) lower risk of CVD mortality in non‐advanced CKM stages. However, for CVD mortality in advanced stages, no significant association was observed with a favorable lifestyle (*p* > 0.05) (Supporting Information S1: eTable 2).

**FIGURE 2:**
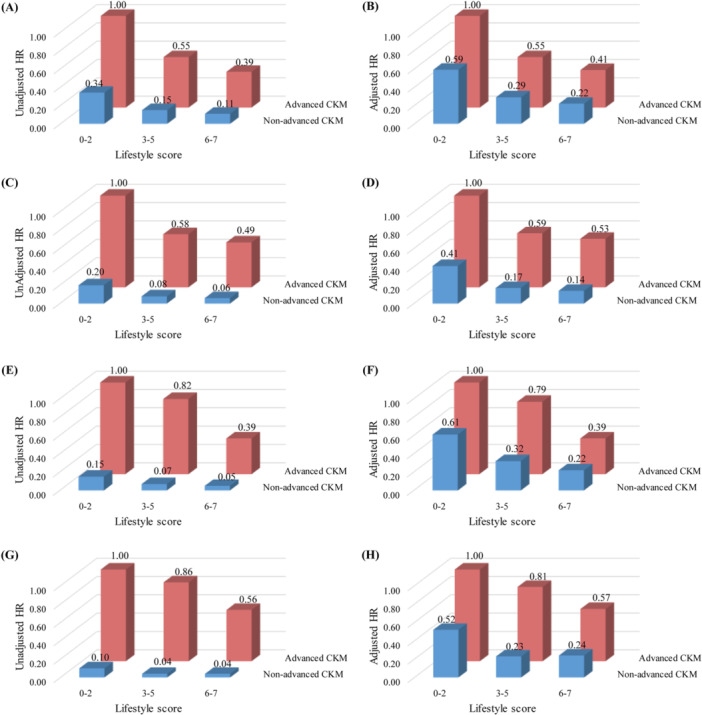
Combined association of lifestyle score and non‐advanced/advanced CKM stages with the risk of mortality Footnote: Adjusted HR was calculated by adjusting age, sex, ethnicity, education, Townsend deprivation index (UK biobank), poverty income ratio (NHANES), body mass index, and history of cancer. (A) and (B), all‐cause mortality in UK Biobank; (C) and (D), cardiovascular mortality in UK Biobank; (E) and (F), all‐cause mortality in NHANES; (G) and (H), cardiovascular mortality in NHANES. CKM, cardiovascular‐kidney‐metabolic; HR, hazard ratio.

Among all combinations of CKM stages and lifestyle group, participants with non‐advanced CKM syndrome and a favorable lifestyle had the lowest risk of all‐cause and CVD mortality. In UK Biobank, the corresponding HRs were 0.22 (95% CI: 0.21, 0.24) for all‐cause mortality and 0.14 (95% CI: 0.12, 0.17) for CVD mortality. In NHANES, the HRs were 0.22 (95% CI: 0.11, 0.42) and 0.24 (95% CI: 0.08, 0.80) for all‐cause and CVD mortality, respectively (Figure 2 and Supporting Information: eTable 3).

The association between lifestyle scores (binary variables) and mortality risk across CKM stages in both cohorts was shown in Supporting Information: eTable 4. Pooled analyses demonstrated that compared to an unfavorable lifestyle, a favorable lifestyle was associated with a reduced risk of all‐cause and CVD mortality in all CKM stages (all *p* < 0.05). Consistent findings were observed using alternative grouping methods (lifestyle score 3–7 vs. 0–2 or lifestyle score 6–7 vs. 0–5).

### 3.4 Subgroup and Sensitivity Analyses

In both databases, higher lifestyle scores were negatively associated with mortality risk across all sex and age subgroups. Sex‐specific analyses revealed a significant interaction only for all‐cause mortality in UK Biobank (*P*_*for interaction*_ = 0.010), while no interaction was observed in NHANES. Age‐stratified analyses showed significant interactions in both cohorts (all *P*_*for interaction*_ < 0.05), with younger participants (< 65 years) experiencing greater risk reductions from a healthy lifestyle compared with older participants (≥ 65 years) (Supporting Information: eTable 5). The association between lifestyle scores and mortality risk remained robust in multiple sensitivity analyses (Supporting Information: eTable 6).

## 4 Discussion

To our knowledge, this is the first study using two national cohorts to investigate the association between a healthy lifestyle and mortality risk across CKM stages. We found a healthy lifestyle was associated with 19% lower all‐cause mortality and 18% lower CVD mortality, with stronger inverse associations in early stages. The strength of association varied across individual healthy lifestyle factors, whereas regular physical activity and low sedentary behavior showed consistent associations. A series of sensitivity analyses supported the robustness of the main findings.

The association between CKM syndrome stages and mortality risk is increasingly discussed [20, 21]. A cohort in China found that the mortality risk in individuals at stage 4 was 3.7 times that in individuals at stage 0 [4]. Confirming and extending these findings, our analysis of UK Biobank and NHANES data revealed significant CKM stage differences in both all‐cause and CVD mortality risk. More importantly, our study demonstrated the inverse association of a healthy lifestyle with mortality risk within the CKM population. Each unit increase in healthy lifestyle score was associated with an 15%–19% reduction in all‐cause mortality, and a 11%–18% reduction in CVD mortality. This aligns with Ning Zhang et al.‘s conclusion that a healthy lifestyle offered protection throughout the longitudinal progression of cardio‐renal metabolic multimorbidity [11]. Furthermore, established benefits of healthy lifestyles for CKM‐related conditions (type 2 diabetes, CVD, CKD) supported our findings [22, 23].

Our analysis revealed varying associations of modifiable lifestyle factors on mortality risk in the CKM population. Specially, both regular physical activity and low sedentary behavior demonstrated statistically significant inverse associations with mortality, persisting after multivariable adjustment. Regular physical activity may reduce mortality risk by promoting an anti‐inflammatory environment, stimulating myocardial regeneration, and mitigating age‐related muscle mass and strength loss [24]. These adaptations collectively improved cardio‐metabolic resilience. Complementarily, low sedentary behavior could exert protection by ameliorating postprandial glycaemia, enhancing insulin sensitivity, and restoring endothelial function [25, 26]. This was corroborated by Han et al.‘s cohort study of type 2 diabetes patients, in which low sedentary behavior was associated with a 10% lower risk in all‐cause mortality [9], indicating that the metabolic and vascular benefits of reduced sedentary behavior ultimately translated into survival gains.

Smoking was a strong independent risk factor for diabetes, CVD, and mortality in previous studies, with proposed mechanisms including oxidative stress and chronic inflammation. Consistent with established evidence, smoking cessation substantially attenuated these excess risks [27]. Our analysis showed that no current smoking was associated with lower all‐cause and CVD mortality in UK Biobank. For alcohol, moderate drinking has been linked to both favorable (higher high density lipoprotein cholesterol concentration and reducing inflammation) and unfavorable (increasing blood pressure) profiles in the literature [28, 29]. In our findings, moderate drinking corresponded to HRs ranging from 0.82 to 0.86 for mortality. A healthy diet and its major food components have been associated with lower mortality in previous studies [30]. For instance, fruits and vegetables contained numerous nutrients and phytochemicals. These compounds have been linked to various physiological markers, including cholesterol levels, blood pressure, inflammation, platelet aggregation, as well as vascular and immune function [31]. We found that a healthy diet was negatively associated with all‐cause mortality in NHANES, though this association did not reach statistical significance for CVD mortality. Finally, both adequate sleep and appropriate social connection were associated with lower mortality risk in UK Biobank, consistent with evidence on the cardio‐metabolic benefits of approximately 7‐h sleep per day [32], and the established health benefits of social connection [33].

Pooled analysis of the two databases revealed that a healthy lifestyle was linked to reduced mortality risk across different stages of CKM syndrome, with more pronounced reductions observed in the early stages. This underscores the potential value of early healthy lifestyle interventions for individuals with CKM syndrome and related chronic conditions. Previous research indicated that diabetes was associated with excess mortality, and the risk increased with the duration of the disease, emphasizing the benefits of early intervention in reducing mortality risk [34]. For individuals in the early stages of diabetes, lifestyle intervention may delay the need for anti‐hyperglycemic drug treatment or induce partial remission of type 2 diabetes [35]. For those in more advanced stages, it may improve blood sugar control and potentially avoid drug intensification [36]. Studies in CKD have similarly demonstrated that early lifestyle intervention can delay disease progression and improve prognosis [37].

We acknowledged several limitations. First, self‐reported lifestyle data may involve recall bias and social desirability bias, which could lead to misclassification of unhealthy habits and affect the estimated associations between healthy lifestyle factors and mortality [38]. Baseline lifestyle information may not fully reflect follow‐up changes, so future research could incorporate repeated measures. Second, despite efforts to harmonize measures, differences existed between cohorts—for example, direct sedentary time reporting in NHANES versus TV‐watching as a proxy in UK Biobank. Third, the lifestyle score assumed equal influence of each factor, and while weighted sensitivity analysis was done, it could not fully capture complex interactions. Fourth, baseline heterogeneity across cohorts was addressed via random‐effects meta‐analysis, but pooled results may lack generalizability. Fifth, unmeasured confounders like genetic predisposition could remain, and we did not account for CKD etiology‐specific mortality risks. Sixth, smoking categorization ignored former smokers’ cardiovascular risks, possibly underestimating the benefits of never smoking [39].

## 5 Conclusions

We found that adherence to a healthy lifestyle was associated with lower mortality risk across all stages of CKM syndrome, with the associations being more pronounced in the early stages. Regular physical activity and low sedentary behavior were consistently associated with lower mortality risk in both cohorts. These findings indicate that promoting a healthy lifestyle (with an emphasis on physical activity and limiting sedentary time) may be related to better long‐term survival outcomes among individuals with CKM syndrome, especially in its early stages.

## Author Contributions

Q.Q.Y., W.F., and M.X. contributed equally to this work and shared first authorship. Conceptualization: S.Y.X., Q.Q.Y., W.F., and M.X. Methodology: S.Y.X., Q.Q.Y., W.F., and M.X. Formal analysis: Q.Q.Y., W.F., and M.X. Writing – original draft: W.F., Q.Q.Y., and M.X. Writing – Review and editing: S.Y.X., Q.Q.Y., W.F., M.X., Y.C.W., A. M. Lett, X.Y.L., and M.L.F. All the authors read and approved of the final paper draft.

## Ethics Statement

Ethical approval for the UK Biobank data was obtained from the North West Multicenter Research Ethics Committee (number: 21/NW/0157). The Institutional Review Board of the National Center for Health Statistics approved the initial survey protocol of the NHANES study. All participants provided informed consent and adhered to the Declaration of Helsinki.

## Conflicts of Interest

The authors declare no conflicts of interest.

## Supporting information


**eFigure 1:** Study flow chart.
**eFigure 2:** Distribution of healthy lifestyle in the study population.
**eTable 1:** Association between cardiovascular‐kidney‐metabolic stages and the risk of mortality.
**eTable 2:** Association between lifestyle score and the risk of mortality in non‐advanced and advanced CKM stages.
**eTable 3:** Combined association of lifestyle score and non‐advanced/advanced CKM stages with the risk of mortality.
**eTable 4:** Pooled results of the association between lifestyle score (binary categorical variable) and the risk of mortality in CKM syndrome.
**eTable 5:** Subgroup analysis of the association between lifestyle score and the risk of mortality in cardiovascular‐kidney‐metabolic syndrome.
**eTable 6:** Summary of sensitivity analysis.

## Data Availability

Datasets from the UK Biobank database can be accessed by contacting the UK Biobank Data Service. Our study was conducted based on a data analysis application (application number: 92014). Datasets from the NHANES database can be accessed at https://www.cdc.gov/nchs/nhanes/index.htm.

## References

[1] S. A. Sebastian , I. Padda , and G. Johal , “Cardiovascular‐Kidney‐Metabolic (CKM) Syndrome: A State‐of‐the‐Art Review,” Current Problems in Cardiology 49, no. 2 (2024): 102344, 10.1016/j.cpcardiol.2023.102344. 38103820

[2] C. E. Ndumele , I. J. Neeland , K. R. Tuttle , et al., “A Synopsis of the Evidence for the Science and Clinical Management of Cardiovascular‐Kidney‐Metabolic (CKM) Syndrome: A Scientific Statement From the American Heart Association,” Circulation 148, no. 20 (2023): 1636–1664, 10.1161/cir.0000000000001186. 37807920

[3] R. Aggarwal , J. W. Ostrominski , and M. Vaduganathan , “Prevalence of Cardiovascular‐Kidney‐Metabolic Syndrome Stages in US Adults, 2011–2020,” Journal of the American Medical Association 331, no. 21 (2024): 1858–1860, 10.1001/jama.2024.6892. 38717747 PMC11079779

[4] N. Li , Y. Li , L. Cui , et al., “Association Between Different Stages of Cardiovascular‐Kidney‐Metabolic Syndrome and the Risk of All‐Cause Mortality,” Atherosclerosis 397 (2024): 118585, 10.1016/j.atherosclerosis.2024.118585. 39255681

[5] S. Abusnana , H. Sabbour , B. Afandi , et al., “Intervening Early in the Cardiovascular‐Kidney‐Metabolic Syndrome: Expert Recommendations From the United Arab Emirates on the Management of Prediabetes,” Vascular Health and Risk Management 22 (2026): 579970, 10.2147/vhrm.S579970. 41783738 PMC12955368

[6] S. Gunnarsson , O. Vito , and R. J. Unwin , “Cardiovascular‐Kidney‐Metabolic Syndrome: Prevalence, Risks, Disease Trajectories, and Early‐Stage Management,” American Journal of Physiology‐Cell Physiology 330, no. 1 (2026): C1–C8, 10.1152/ajpcell.00499.2025. 41269265

[7] S. Patiño‐Cardona , C. Pascual‐Morena , I. Sequí‐Domínguez , et al., “Safety of Metformin in Chronic Kidney Disease and Type 2 Diabetes Mellitus: A Systematic Review and Meta‐Analysis,” European Journal of Pharmacology 1015 (2026): 178560, 10.1016/j.ejphar.2026.178560. 41548682

[8] M. Niu , J. Chen , R. Hou , et al., “Emerging Healthy Lifestyle Factors and All‐Cause Mortality Among People With Metabolic Syndrome and Metabolic Syndrome‐Like Characteristics in NHANES,” Journal of Translational Medicine 21, no. 1 (2023): 239, 10.1186/s12967-023-04062-1. 37005663 PMC10068159

[9] H. Han , Y. Cao , C. Feng , et al., “Association of a Healthy Lifestyle With All‐Cause and Cause‐Specific Mortality Among Individuals With Type 2 Diabetes: A Prospective Study in UK Biobank,” Diabetes Care 45, no. 2 (2022): 319–329, 10.2337/dc21-1512. 34857534

[10] H. Larkin , “Here's What to Know About Cardiovascular‐Kidney‐Metabolic Syndrome, Newly Defined by the AHA,” Journal of the American Medical Association 330, no. 21 (2023): 2042, 10.1001/jama.2023.22276. 37938824

[11] N. Zhang , X. Liu , L. Wang , et al., “Lifestyle Factors and Their Relative Contributions to Longitudinal Progression of Cardio‐Renal‐Metabolic Multimorbidity: A Prospective Cohort Study,” Cardiovascular Diabetology 23, no. 1 (2024): 265, 10.1186/s12933-024-02347-3. 39026309 PMC11264843

[12] C. Sudlow , J. Gallacher , N. Allen , et al., “UK Biobank: An Open Access Resource for Identifying the Causes of a Wide Range of Complex Diseases of Middle and Old Age,” PLoS Medicine 12, no. 3 (2015): e1001779, 10.1371/journal.pmed.1001779. 25826379 PMC4380465

[13] J. D. Bundy , K. T. Mills , H. He , et al., “Social Determinants of Health and Premature Death Among Adults in the USA From 1999 to 2018: A National Cohort Study,” Lancet Public Health 8, no. 6 (2023): e422–e431, 10.1016/s2468-2667(23)00081-6. 37244672 PMC10349537

[14] Q. Zheng , Z. Cao , J. Teng , Q. Lu , P. Huang , and J. Zhou , “Association Between Atherogenic Index of Plasma With All‐Cause and Cardiovascular Mortality in Individuals With Cardiovascular‐Kidney‐Metabolic Syndrome,” Cardiovascular Diabetology 24, no. 1 (2025): 183, 10.1186/s12933-025-02742-4. 40287685 PMC12034140

[15] C. Jia , Y. Zeng , X. Huang , et al., “Lifestyle Patterns, Genetic Susceptibility, and Risk of Valvular Heart Disease: A Prospective Cohort Study Based on the UK Biobank,” European Journal of Preventive Cardiology 30, no. 15 (2023): 1665–1673, 10.1093/eurjpc/zwad177. 37259902

[16] Y.‐B. Zhang , C. Chen , X.‐F. Pan , et al., “Associations of Healthy Lifestyle and Socioeconomic Status With Mortality and Incident Cardiovascular Disease: Two Prospective Cohort Studies,” BMJ 373 (2021): n604, 10.1136/bmj.n604. 33853828 PMC8044922

[17] C. Wang , S. I. Bangdiwala , S. Rangarajan , et al., “Association of Estimated Sleep Duration and Naps With Mortality and Cardiovascular Events: A Study of 116 632 People From 21 Countries,” European Heart Journal 40, no. 20 (2019): 1620–1629, 10.1093/eurheartj/ehy695. 30517670 PMC6528160

[18] Y. J. van de Vegte , M. A. Said , M. Rienstra , P. van der Harst , and N. Verweij , “Genome‐Wide Association Studies and Mendelian Randomization Analyses for Leisure Sedentary Behaviours,” Nature Communications 11, no. 1 (2020): 1770, 10.1038/s41467-020-15553-w. PMC7174427 32317632

[19] Z. Zhang , “Multiple Imputation With Multivariate Imputation by Chained Equation (MICE) Package,” Annals of Translational Medicine 4, no. 2 (2016): 30, 10.3978/j.issn.2305-5839.2015.12.63. 26889483 PMC4731595

[20] H. Ji , C. Sabanayagam , K. Matsushita , et al., “Sex Differences in Cardiovascular‐Kidney‐Metabolic Syndrome: 30‐Year US Trends and Mortality Risks‐Brief Report,” Arteriosclerosis, Thrombosis, and Vascular Biology 45, no. 1 (2025): 157–161, 10.1161/atvbaha.124.321629. 39665141 PMC11729504

[21] S. E. Claudel , I. M. Schmidt , S. S. Waikar , et al., “Cumulative Incidence of Mortality Associated With Cardiovascular–Kidney–Metabolic (CKM) Syndrome,” Journal of the American Society of Nephrology 36 (2015): 1343–1351, 10.1681/asn.0000000637. PMC12187233 39932805

[22] T. Geng , K. Zhu , Q. Lu , et al., “Healthy Lifestyle Behaviors, Mediating Biomarkers, and Risk of Microvascular Complications Among Individuals With Type 2 Diabetes: A Cohort Study,” PLoS Medicine 20, no. 1 (2023): e1004135, 10.1371/journal.pmed.1004135. 36626356 PMC9831321

[23] A. C. Ricardo , C. A. Anderson , W. Yang , et al., “Healthy Lifestyle and Risk of Kidney Disease Progression, Atherosclerotic Events, and Death in CKD: Findings From the Chronic Renal Insufficiency Cohort (CRIC) Study,” American Journal of Kidney Diseases 65, no. 3 (2015): 412–424, 10.1053/j.ajkd.2014.09.016. 25458663 PMC4339665

[24] C. Fiuza‐Luces , A. Santos‐Lozano , M. Joyner , et al., “Exercise Benefits in Cardiovascular Disease: Beyond Attenuation of Traditional Risk Factors,” Nature Reviews Cardiology 15, no. 12 (2018): 731–743, 10.1038/s41569-018-0065-1. 30115967

[25] J. T. Gale , D. L. Wei , J. J. Haszard , R. C. Brown , R. W. Taylor , and M. C. Peddie , “Breaking Up Evening Sitting With Resistance Activity Improves Postprandial Glycemic Response: A Randomized Crossover Study,” Medicine & Science in Sports & Exercise 55, no. 8 (2023): 1471–1480, 10.1249/mss.0000000000003166. 36921112 PMC10348652

[26] J. Henson , M. J. Davies , D. H. Bodicoat , et al., “Breaking Up Prolonged Sitting With Standing or Walking Attenuates the Postprandial Metabolic Response in Postmenopausal Women: A Randomized Acute Study,” Diabetes Care 39, no. 1 (2016): 130–138, 10.2337/dc15-1240. 26628415

[27] U. Mons , A. Muezzinler , C. Gellert , et al., “Impact of Smoking and Smoking Cessation on Cardiovascular Events and Mortality Among Older Adults: Meta‐Analysis of Individual Participant Data From Prospective Cohort Studies of the CHANCES Consortium,” BMJ 350, no. apr20 2 (2015): h1551, 10.1136/bmj.h1551. 25896935 PMC4413837

[28] P. E. Ronksley , S. E. Brien , B. J. Turner , K. J. Mukamal , and W. A. Ghali , “Association of Alcohol Consumption With Selected Cardiovascular Disease Outcomes: A Systematic Review and Meta‐Analysis,” BMJ 342, no. feb22 1 (2011): d671, 10.1136/bmj.d671. 21343207 PMC3043109

[29] I. Y. Millwood , R. G. Walters , X. W. Mei , et al., “Conventional and Genetic Evidence on Alcohol and Vascular Disease Aetiology: A Prospective Study of 500 000 Men and Women in China,” Lancet 393, no. 10183 (2019): 1831–1842, 10.1016/s0140-6736(18)31772-0. 30955975 PMC6497989

[30] Z. Shan , F. Wang , Y. Li , et al., “Healthy Eating Patterns and Risk of Total and Cause‐Specific Mortality,” JAMA Internal Medicine 183, no. 2 (2023): 142–153, 10.1001/jamainternmed.2022.6117. 36622660 PMC9857813

[31] D. Aune , E. Giovannucci , P. Boffetta , et al., “Fruit and Vegetable Intake and the Risk of Cardiovascular Disease, Total Cancer and All‐Cause Mortality‐A Systematic Review and Dose‐Response Meta‐Analysis of Prospective Studies,” International Journal of Epidemiology 46, no. 3 (2017): 1029–1056, 10.1093/ije/dyw319. 28338764 PMC5837313

[32] J. Yin , X. Jin , Z. Shan , et al., “Relationship of Sleep Duration With All‐Cause Mortality and Cardiovascular Events: A Systematic Review and Dose‐Response Meta‐Analysis of Prospective Cohort Studies,” Journal of the American Heart Association 6, no. 9 (2017): e005947, 10.1161/jaha.117.005947. 28889101 PMC5634263

[33] G. Mahalingam , S. Samtani , B. C. P. Lam , et al., “Social Connections and Risk of Incident Mild Cognitive Impairment, Dementia, and Mortality in 13 Longitudinal Cohort Studies of Ageing,” Alzheimer's & Dementia 19, no. 11 (2023): 5114–5128, 10.1002/alz.13072. PMC10603208 37102417

[34] O. Tang , K. Matsushita , J. Coresh , et al., “Mortality Implications of Prediabetes and Diabetes in Older Adults,” Diabetes Care 43, no. 2 (2020): 382–388, 10.2337/dc19-1221. 31776141 PMC6971785

[35] E. W. Gregg , H. Chen , L. E. Wagenknecht , et al., “Association of an Intensive Lifestyle Intervention With Remission of Type 2 Diabetes,” Journal of the American Medical Association 308, no. 23 (2012): 2489, 10.1001/jama.2012.67929. 23288372 PMC4771522

[36] K. Kempf , B. Altpeter , J. Berger , et al., “Efficacy of the Telemedical Lifestyle Intervention Program TeLiPro in Advanced Stages of Type 2 Diabetes: A Randomized Controlled Trial,” Diabetes Care 40, no. 7 (2017): 863–871, 10.2337/dc17-0303. 28500214

[37] E. A. Hu , J. Coresh , C. A. M. Anderson , et al., “Adherence to Healthy Dietary Patterns and Risk of CKD Progression and All‐Cause Mortality: Findings From the CRIC (Chronic Renal Insufficiency Cohort) Study,” American Journal of Kidney Diseases 77, no. 2 (2021): 235–244, 10.1053/j.ajkd.2020.04.019. 32768632 PMC7855760

[38] H. Xie , J. Li , X. Zhu , et al., “Association Between Healthy Lifestyle and the Occurrence of Cardiometabolic Multimorbidity in Hypertensive Patients: A Prospective Cohort Study of UK Biobank,” Cardiovascular Diabetology 21, no. 1 (2022): 199, 10.1186/s12933-022-01632-3. 36183084 PMC9526960

[39] K. K. Teo , S. Ounpuu , S. Hawken , et al., “Tobacco Use and Risk of Myocardial Infarction in 52 Countries in the INTERHEART Study: A Case‐Control Study,” Lancet 368, no. 9536 (2006): 647–658, 10.1016/s0140-6736(06)69249-0. 16920470

