# Supplementary material for: Associations of Healthy Lifestyle With All‐Cause and Cardiovascular Disease Mortality in Individuals With Cardiovascular‐Kidney‐Metabolic Syndrome: Two Prospective Cohort Studies
Source: Clin Cardiol. 2026 Aug 5;49(8):e70436. doi: 10.1002/clc.70436 (PMC13439629; doi:10.1002/clc.70436)
Supplement: Supplementary file 1 [file CLC-49-e70436-s001.docx]

**Supplementary Materials**

**List of supplementary materials**

**eMethods**

**eTable 1** Association between cardiovascular-kidney-metabolic stages and the risk of mortality

**eTable 2** Association between lifestyle score and the risk of mortality in non-advanced and advanced CKM stages

**eTable 3** Combined association of lifestyle score and non-advanced/advanced CKM stages with the risk of mortality

**eTable 4** Pooled results of the association between lifestyle score (binary categorical variable) and the risk of mortality in CKM syndrome

**eTable 5** Subgroup analysis of the association between lifestyle score and the risk of mortality in cardiovascular-kidney-metabolic syndrome

**eTable 6** Summary of sensitivity analysis

**eFigure 1** Study flow chart

**eFigure 2** Distribution of healthy lifestyle in the study population

**eMethods**

***1. Definition of CKM*** ***stages 0-4***

**Stage 0** involves individuals without overweight/obesity, metabolic risk factors (hypertension, hypertriglyceridemia, metabolic syndrome, diabetes), CKD, or subclinical/clinical CVD;

**Stage 1** included individuals with overweight/obesity, abdominal obesity, or dysfunctional adipose tissue without other metabolic risk factors or CKD: body mass index ≥ 25 kg/m^2^, waist circumference ≥ 88/102 cm female/male, and/or fasting glucose ≥ 100-124 mg/dl (only for US NHANES) or glycated hemoglobin between 5.7% and 6.4%;

**Stage 2** included individuals with metabolic risk factors or CKD;

**Stage 3** included patients with subclinical atherosclerotic CVD or subclinical heart failure who had excessive/dysfunctional obesity, other metabolic risk factors, or CKD. We used very high-risk Kidney Disease Improving Global Outcomes CKD stages (stage G4 or G5 CKD) **(eTable m1)** and a high-predicted 10-year CVD risk by the AHA Predicting Risk of CVD EVENTs (PREVENT) equations (base model) as risk equivalents for subclinical CVD ^1,2^.

**eTable m1** GFR categories in CKD ^3^

| **GFR category** | **GFR (ml/min per 1.73 m^2^)** | **Terms** |
| --- | --- | --- |
| G1 | ≥ 90 | Normal or high |
| G2 | 60-89 | Mildly decreased |
| G3a | 45-59 | Mildly to moderately decreased |
| G3b | 30-44 | Moderately to severely decreased |
| G4 | 15-29 | Severely decreased |
| G5 | < 15 | Kidney failure |

CKD, chronic kidney disease; GFR, glomerular filtration rate

High-predicted 10-year CVD risk was defined as ≥ 20% 10-year CVD risk by the American Heart Association Predicting Risk of CVD EVENTs (PREVENT) equations. The PREVENT equation ^4^ was developed and validated for adults aged 30-79 years old. In the UK Biobank, the baseline age range of participants was 40-69 years, and the 10-year CVD risk was calculated for all study population. In the US NHANES, the 10-year CVD risk was not estimated for adults aged < 30 years. To minimize underestimation of CKD Stage 3, adults aged ≥ 80 years were assigned an age of 79 years for the calculation of 10-year CVD risks ^2^. In addition, the PREVENT equation was developed for variables with specific bounds **(eTable m2)**. To approximate PREVENT risk strata, values for these variables above or below these bounds were set to the upper or lower bounds of allowable values respectively ^2^.

**eTable m2** Variable bounds in the PREVENT equation ^4^

| **Variables** | **Lower bounds** | **Upper bounds** |
| --- | --- | --- |
| Total cholesterol, mg/dl | 130 | 320 |
| High density lipoprotein cholesterol, mg/dl | 20 | 100 |
| Systolic blood pressure, mmHg | 90 | 200 |
| Estimated glomerular filtration rate, ml/min per 1.73 m^2^ | 15 | 140 |
| Urinary albumin creatinine ratio, mg/g | 0 | 25000 |
| Glycated hemoglobin, % | 3 | 15 |

**Stage 4** includes patients with clinical CVD (coronary heart disease, heart failure, stroke, peripheral artery disease, atrial fibrillation) who had excessive/dysfunctional obesity, other metabolic risk factors, or CKD.

***2. Assessment of lifestyle factors***

The assessments of healthy diet in the UK Biobank and NHANES were shown in **eTable m3**.

**eTable m3** Assessments of healthy diet in the UK Biobank and NHANES

| **UK Biobank: a low-risk diet was defined as consuming at least half of the 10 recommended food groups ^5^** | | | | | |
| --- | --- | --- | --- | --- | --- |
| Diet component | Intake goal | | | Amount per serving | |
| Fruit | ≥ 3 servings/day | | | 1309 -1 piece  1319 -5 pieces | |
| Vegetable | ≥ 3 servings/day | | | 1289/1299 -3 heaped tablespoons | |
| Whole grains | ≥ 3 servings/day | | | 1438/1448 (wholemeal/wholegrain bread slices) -1 slice/day  1458/1468 (bran/oat/muesli cereal bowls) -1 bowl/day | |
| (Shell)fish | ≥ 2 servings/week | | | 1329/1339 -once/week | |
| Dairy | ≥ 2 servings/day | | | 1408 -1 piece/day  1418 -1 glass/day if consumption of anytype of milk | |
| Vegetable oils | ≥ 2 servings/day | | | 1428 (Flora Pro-Active/Benecol spread) /2654 (Flora Pro-Active/Benecol, soft margarine, olive oil based, polyunsaturated/sunflower oil based, other low or reduced fat spread) -1 serving/day if in combination with eating at least 2 slices of bread (ID 1438) | |
| Refined grains | ≤ 2 servings/day | | | 1438/1448 (white, brown, other bread) -1 slice/day  1458/1468 (biscuit, other cereals) -1 bowl/day | |
| Processed meats | ≤ 1 serving/week | | | 1349 -once/day | |
| Unprocessed meats | ≤ 2 servings/week | | | 1359/1369/1379/1389-once/week | |
| Sugar-sweetened beverages | Don't drink | | | 6144 -only 0 serving was possible here | |
| **NHANES: a low-risk diet was defined as being in the top two quintiles of the Healthy Eating Index ^6,7^** | | | | | |
| Component | | Maximum points | Standard for maximum score | | Standard for minimum score of zero |
| Adequacy: | | | | | |
| Total Fruits | | 5 | ≥ 0.8 cup equiv. per 1,000 kcal | | No Fruits |
| Whole Fruits | | 5 | ≥ 0.4 cup equiv. per 1,000 kcal | | No Whole Fruits |
| Total Vegetables | | 5 | ≥ 1.1 cup equiv. per 1,000 kcal | | No Vegetables |
| Greens and Beans | | 5 | ≥ 0.2 cup equiv. per 1,000 kcal | | No Dark Green Vegetables or Legumes |
| Whole Grains | | 10 | ≥ 1.5 oz equiv. per 1,000 kcal | | No Whole Grains |
| Dairy | | 10 | ≥ 1.3 cup equiv. per 1,000 kcal | | No Dairy |
| Total Protein Foods | | 5 | ≥ 2.5 oz equiv. per 1,000 kcal | | No Protein Foods |
| Seafood and Plant Proteins | | 5 | ≥ 0.8 oz equiv. per 1,000 kcal | | No Seafood or Plant Proteins |
| Fatty Acids | | 10 | (PUFAS+ MUFAS)/SFAs ≥ 2.5 | | (PUFAS+ MUFAS)/SFAs ≤ 1.2 |
| Moderation: | | | | | |
| Refined Grains | | 10 | ≤ 1.8 oz equiv. per 1,000 kcal | | ≥ 4.3 oz equiv. per 1,000 kcal |
| Sodium | | 10 | ≤ 1.1 gram per 1,000 kcal | | ≥ 2.0 gram per 1,000 kcal |
| Added sugars | | 10 | ≤ 6.5% of energy | | ≥ 26% of energy |
| Saturated Fats | | 10 | ≤ 8% of energy | | ≥ 16% of energy |

PUFA, polyunsaturated fatty acid; MUFA, monounsaturated fatty acid; SFA, saturated fatty acid

**Reference**

1. Ndumele CE, Neeland IJ, Tuttle KR, et al.; American Heart Association. A Synopsis of the Evidence for the Science and Clinical Management of Cardiovascular-Kidney-Metabolic (CKM) Syndrome: A Scientific Statement From the American Heart Association. Circulation. 2023 Nov 14;148(20):1636-1664.
2. Aggarwal R, Ostrominski JW, Vaduganathan M. Prevalence of Cardiovascular-Kidney-Metabolic Syndrome Stages in US Adults, 2011-2020. JAMA. 2024 Jun 4;331(21):1858-1860.
3. Kidney Disease: Improving Global Outcomes (KDIGO) CKD Work Group. KDIGO 2024 Clinical Practice Guideline for the Evaluation and Management of Chronic Kidney Disease. Kidney Int. 2024 Apr;105(4S):S117-S314.
4. PREVENTTM Online Calculator. <https://professional.heart.org/en/guidelines%20and-statements/prevent-calculator>.
5. Han H, Cao Y, Feng C, et al. Association of a Healthy Lifestyle With All-Cause and Cause-Specific Mortality Among Individuals With Type 2 Diabetes: A Prospective Study in UK Biobank. Diabetes Care. 2022 Feb 1;45(2):319-329.
6. Developing the HEI Components and Scoring Standards. https://epi.grants.cancer.gov/hei/developing.html#2015.
7. Zhang YB, Chen C, Pan XF, et al. Associations of healthy lifestyle and socioeconomic status with mortality and incident cardiovascular disease: two prospective cohort studies. BMJ. 2021 Apr 14;373:n604.

**eTable 1** Association between cardiovascular-kidney-metabolic stages and the risk of mortality

|  | **All-cause mortality** | | **Cardiovascular mortality** | |
| --- | --- | --- | --- | --- |
|  | **Model 0** | **Model 1** | **Model 0** | **Model 1** |
| **UK Biobank** |  |  |  |  |
| Stage 0 | 1.00 (Ref) | 1.00 (Ref) | 1.00 (Ref) | 1.00 (Ref) |
| Stage 1 | 1.22 (1.13, 1.31) *** | 1.00 (0.92, 1.07) | 1.19 (0.94, 1.49) | 0.86 (0.68, 1.08) |
| Stage 2 | 2.07 (1.96, 2.19) *** | 1.13 (1.07, 1.20) *** | 3.27 (2.77, 3.87) *** | 1.50 (1.26, 1.78) *** |
| Stage 3 | 8.50 (7.85, 9.21) *** | 2.03 (1.86, 2.21) *** | 20.18 (16.49, 24.70) *** | 3.55 (2.86, 4.41) *** |
| Stage 4 | 6.34 (5.97, 6.74) *** | 2.09 (1.96, 2.24) *** | 19.80 (16.70, 23.48) *** | 5.02 (4.19, 6.03) *** |
| **NHANES 2007-2018** | | | | |
| Stage 0 | 1.00 (Ref) | 1.00 (Ref) | 1.00 (Ref) | 1.00 (Ref) |
| Stage 1 | 1.07 (0.61, 1.90) | 0.82 (0.46, 1.46) | 3.12 (0.69, 14.08) | 2.35 (0.52, 10.69) |
| Stage 2 | 2.30 (1.42, 3.73) *** | 0.90 (0.55, 1.49) | 4.16 (1.00, 17.28) * | 1.36 (0.32, 5.70) |
| Stage 3 | 19.52 (12.14, 31.38) *** | 1.61 (0.96, 2.69) | 54.49 (13.44, 220.94) *** | 2.79 (0.67, 11.69) |
| Stage 4 | 20.31 (12.61, 32.71) *** | 2.29 (1.37, 3.82) ** | 76.76 (18.97, 310.55) *** | 5.81 (1.40, 24.17) * |

Model 0: unadjusted; Model 1: adjusted for age, sex, ethnicity, education, Townsend deprivation index (UK biobank), poverty income ratio (NHANES), lifestyle score, body mass index, and history of cancer. * *P* < 0.05, ** *P* < 0.01, *** *P* < 0.001

**eTable 2** Association between lifestyle score and the risk of mortality in non-advanced and advanced CKM stages

|  | **All-cause mortality** | | **Cardiovascular mortality** | |
| --- | --- | --- | --- | --- |
|  | **Model 0** | **Model 1** | **Model 0** | **Model 1** |
| **Non-advanced CKM stages (stages 0-2)** | | | | |
| **UK Biobank** |  |  |  |  |
| Lifestyle score |  |  |  |  |
| 0-2 | 1.00 (Ref) | 1.00 (Ref) | 1.00 (Ref) | 1.00 (Ref) |
| 3-5 | 0.45 (0.43, 0.47) *** | 0.48 (0.46, 0.51) *** | 0.39 (0.34, 0.43) *** | 0.42 (0.37, 0.47) *** |
| 6-7 | 0.34 (0.32, 0.36) *** | 0.38 (0.36, 0.40) *** | 0.31 (0.27, 0.36) *** | 0.35 (0.30, 0.41) *** |
| **NHANES 2007-2018** | | | | |
| Lifestyle score |  |  |  |  |
| 0-2 | 1.00 (Ref) | 1.00 (Ref) | 1.00 (Ref) | 1.00 (Ref) |
| 3-5 | 0.45 (0.34, 0.60) *** | 0.52 (0.39, 0.70) *** | 0.37 (0.21, 0.68) ** | 0.43 (0.23, 0.79) ** |
| 6-7 | 0.32 (0.16, 0.61) ** | 0.36 (0.18, 0.71) ** | 0.40 (0.12, 1.38) | 0.43 (0.12, 1.53) |
| **Advanced CKM stages (stages 3-4)** | | | | |
| **UK Biobank** |  |  |  |  |
| Lifestyle score |  |  |  |  |
| 0-2 | 1.00 (Ref) | 1.00 (Ref) | 1.00 (Ref) | 1.00 (Ref) |
| 3-5 | 0.55 (0.51, 0.59) *** | 0.56 (0.52, 0.61) *** | 0.58 (0.51, 0.66) *** | 0.60 (0.52, 0.68) *** |
| 6-7 | 0.39 (0.35, 0.43) *** | 0.41 (0.37, 0.46) *** | 0.50 (0.42, 0.59) *** | 0.53 (0.44, 0.64) *** |
| **NHANES 2007-2018** | | | | |
| Lifestyle score |  |  |  |  |
| 0-2 | 1.00 (Ref) | 1.00 (Ref) | 1.00 (Ref) | 1.00 (Ref) |
| 3-5 | 0.82 (0.69, 0.97) * | 0.78 (0.66, 0.93) ** | 0.86 (0.65, 1.14) | 0.81 (0.60, 1.09) |
| 6-7 | 0.39 (0.21, 0.74) ** | 0.39 (0.21, 0.75) ** | 0.56 (0.22, 1.38) | 0.59 (0.23, 1.49) |
|  |  |  |  |  |

Model 0: unadjusted; Model 1: adjusted for age, sex, ethnicity, education, Townsend deprivation index (UK biobank), poverty income ratio (NHANES), body mass index, and history of cancer. CKM, cardiovascular-kidney-metabolic. * *P* < 0.05, ** *P* < 0.01, *** *P* < 0.001

**eTable 3** Combined association of lifestyle score and non-advanced/advanced CKM stages with the risk of mortality

|  | **All-cause mortality** | | **Cardiovascular mortality** | |
| --- | --- | --- | --- | --- |
|  | **Model 0** | **Model 1** | **Model 0** | **Model 1** |
| **UK Biobank** | | | | |
| Advanced CKM+lifestyle score 0-2 | 1.00 (Ref) | 1.00 (Ref) | 1.00 (Ref) | 1.00 (Ref) |
| Advanced CKM+lifestyle score 3-5 | 0.55 (0.51, 0.59) *** | 0.55 (0.51, 0.59) *** | 0.58 (0.51, 0.66) *** | 0.59 (0.52, 0.67) *** |
| Advanced CKM+lifestyle score 6-7 | 0.39 (0.35, 0.43) *** | 0.41 (0.37, 0.45) *** | 0.49 (0.41, 0.59) *** | 0.53 (0.44, 0.63) *** |
| Non-advanced CKM+lifestyle score 0-2 | 0.34 (0.31, 0.36) *** | 0.59 (0.55, 0.64) *** | 0.20 (0.17, 0.24) *** | 0.41 (0.35, 0.48) *** |
| Non-advanced CKM+lifestyle score 3-5 | 0.15 (0.14, 0.16) *** | 0.29 (0.27, 0.31) *** | 0.08 (0.07, 0.09) *** | 0.17 (0.15, 0.19) *** |
| Non-advanced CKM+lifestyle score 6-7 | 0.11 (0.11, 0.12) *** | 0.22 (0.21, 0.24) *** | 0.06 (0.05, 0.07) *** | 0.14 (0.12, 0.17) *** |
| **NHANES 2007-2018** | | | | |
| Advanced CKM+lifestyle score 0-2 | 1.00 (Ref) | 1.00 (Ref) | 1.00 (Ref) | 1.00 (Ref) |
| Advanced CKM+lifestyle score 3-5 | 0.82 (0.69, 0.97) * | 0.79 (0.66, 0.93) ** | 0.86 (0.65, 1.14) | 0.81 (0.60, 1.08) |
| Advanced CKM+lifestyle score 6-7 | 0.39 (0.21, 0.74) ** | 0.39 (0.20, 0.74) ** | 0.56 (0.23, 1.38) | 0.57 (0.23, 1.43) |
| Non-advanced CKM+lifestyle score 0-2 | 0.15 (0.11, 0.19) *** | 0.61 (0.45, 0.81) ** | 0.10 (0.06, 0.17) *** | 0.52 (0.29, 0.92) * |
| Non-advanced CKM+lifestyle score 3-5 | 0.07 (0.05, 0.08) *** | 0.32 (0.25, 0.41) *** | 0.04 (0.02, 0.06) *** | 0.23 (0.14, 0.37) *** |
| Non-advanced CKM+lifestyle score 6-7 | 0.05 (0.02, 0.09) *** | 0.22 (0.11, 0.42) *** | 0.04 (0.01, 0.13) *** | 0.24 (0.08, 0.80) * |

Model 0: unadjusted; Model 1: adjusted for age, sex, ethnicity, education, Townsend deprivation index (UK biobank), poverty income ratio (NHANES), body mass index, and history of cancer. CKM, cardiovascular-kidney-metabolic. * *P* < 0.05, ** *P* < 0.01, *** *P* < 0.001

**eTable 4** Pooled results of the association between lifestyle score (binary categorical variable) and the risk of mortality in CKM syndrome

|  | **Adjusted hazard ratio (95% confidence interval)** | | |
| --- | --- | --- | --- |
|  | **UK Biobank** | **NHANES 2007-2018** | **Pooled** |
| **All-cause mortality** |  |  |  |
| **Non-advanced CKM stages (stages 0-2)** | | | |
| lifestyle score (3-7 vs 0-2) | 0.46 (0.44, 0.49) *** | 0.51 (0.39, 0.68) *** | 0.46 (0.44, 0.49) |
| lifestyle score (6-7 vs 0-5) | 0.73 (0.70, 0.77) *** | 0.59 (0.31, 1.13) | 0.73 (0.69, 0.76) |
| lifestyle score (6-7 vs 0-2) | 0.37 (0.35, 0.40) *** | 0.35 (0.17, 0.70) ** | 0.37 (0.34, 0.39) |
| **Advanced CKM stages (stages 3-4)** | | | |
| lifestyle score (3-7 vs 0-2) | 0.54 (0.50, 0.58) *** | 0.77 (0.64, 0.91) ** | 0.65 (0.42, 0.87) |
| lifestyle score (6-7 vs 0-5) | 0.68 (0.62, 0.74) *** | 0.47 (0.25, 0.89) * | 0.67 (0.61, 0.73) |
| lifestyle score (6-7 vs 0-2) | 0.40 (0.36, 0.45) *** | 0.39 (0.20, 0.77) ** | 0.40 (0.36, 0.44) |
| **CKM stages 0-4** |  |  |  |
| lifestyle score (3-7 vs 0-2) | 0.49 (0.47, 0.51) *** | 0.70 (0.61, 0.82) *** | 0.59 (0.38, 0.79) |
| lifestyle score (6-7 vs 0-5) | 0.73 (0.70, 0.75) *** | 0.53 (0.34, 0.83) ** | 0.67 (0.49, 0.85) |
| lifestyle score (6-7 vs 0-2) | 0.38 (0.36, 0.40) *** | 0.37 (0.23, 0.61) *** | 0.38 (0.36, 0.40) |
|  |  |  |  |
| **Cardiovascular mortality** |  |  |  |
| **Non-advanced CKM stages (stages 0-2)** | | | |
| lifestyle score (3-7 vs 0-2) | 0.41 (0.36, 0.46) *** | 0.43 (0.24, 0.79) ** | 0.41 (0.36, 0.46) |
| lifestyle score (6-7 vs 0-5) | 0.77 (0.69, 0.86) *** | 0.82 (0.25, 2.70) | 0.77 (0.69, 0.86) |
| lifestyle score (6-7 vs 0-2) | 0.35 (0.30, 0.41) *** | 0.31 (0.08, 1.14) | 0.35 (0.29, 0.40) |
| **Advanced CKM stages (stages 3-4)** | | | |
| lifestyle score (3-7 vs 0-2) | 0.59 (0.51, 0.67) *** | 0.80 (0.60, 1.08) | 0.66 (0.47, 0.86) |
| lifestyle score (6-7 vs 0-5) | 0.83 (0.72, 0.96) * | 0.69 (0.28, 1.71) | 0.83 (0.71, 0.94) |
| lifestyle score (6-7 vs 0-2) | 0.54 (0.44, 0.66) *** | 0.64 (0.24, 1.68) | 0.54 (0.43, 0.65) |
| **CKM stages 0-4** |  |  |  |
| lifestyle score (3-7 vs 0-2) | 0.48 (0.44, 0.52) *** | 0.74 (0.56, 0.96) * | 0.59 (0.34, 0.84) |
| lifestyle score (6-7 vs 0-5) | 0.79 (0.73, 0.87) *** | 0.75 (0.37, 1.53) | 0.79 (0.72, 0.86) |
| lifestyle score (6-7 vs 0-2) | 0.41 (0.36, 0.47) *** | 0.52 (0.24, 1.12) | 0.41 (0.36, 0.47) |

Adjusted for age, sex, ethnicity, education, Townsend deprivation index (UK biobank), poverty income ratio (NHANES), body mass index, and history of cancer (for CKM stages 0-4, additionally adjusting for CKM stage). CKM, cardiovascular-kidney-metabolic. * *P* < 0.05, ** *P* < 0.01, *** *P* < 0.001

**eTable 5** Subgroup analysis of the association between lifestyle score and the risk of mortality in cardiovascular-kidney-metabolic syndrome

| **Subgroup** | **UK Biobank** | | **NHANES 2007-2018** | |
| --- | --- | --- | --- | --- |
|  | **All-cause mortality** | **Cardiovascular mortality** | **All-cause mortality** | **Cardiovascular mortality** |
| **Males** |  |  |  |  |
| Lifestyle score |  |  |  |  |
| 0-2 | 1.00 (Ref) | 1.00 (Ref) | 1.00 (Ref) | 1.00 (Ref) |
| 3-5 | 0.50 (0.47, 0.53) *** | 0.50 (0.45, 0.56) *** | 0.71 (0.58, 0.87) ** | 0.82 (0.57, 1.19) |
| 6-7 | 0.37 (0.35, 0.40) *** | 0.43 (0.37, 0.49) *** | 0.32 (0.16, 0.61) ** | 0.58 (0.22, 1.50) |
| **Females** |  |  |  |  |
| Lifestyle score |  |  |  |  |
| 0-2 | 1.00 (Ref) | 1.00 (Ref) | 1.00 (Ref) | 1.00 (Ref) |
| 3-5 | 0.51 (0.48, 0.55) *** | 0.47 (0.40, 0.55) *** | 0.74 (0.59, 0.93) * | 0.66 (0.44, 0.98) * |
| 6-7 | 0.42 (0.39, 0.46) *** | 0.41 (0.33, 0.51) *** | 0.61 (0.32, 1.18) | 0.76 (0.23, 2.54) |
| ***P* _for interaction_** | 0.010 | 0.592 | 0.306 | 0.606 |
| **Age < 65 years** |  |  |  |  |
| Lifestyle score |  |  |  |  |
| 0-2 | 1.00 (Ref) | 1.00 (Ref) | 1.00 (Ref) | 1.00 (Ref) |
| 3-5 | 0.50 (0.47, 0.52) *** | 0.46 (0.41, 0.52) *** | 0.58 (0.42, 0.80) ** | 0.48 (0.25, 0.94) * |
| 6-7 | 0.38 (0.36, 0.41) *** | 0.39 (0.34, 0.45) *** | 0.18 (0.04, 0.73) * | - |
| **Age ≥ 65 years** |  |  |  |  |
| Lifestyle score |  |  |  |  |
| 0-2 | 1.00 (Ref) | 1.00 (Ref) | 1.00 (Ref) | 1.00 (Ref) |
| 3-5 | 0.57 (0.53, 0.61) *** | 0.61 (0.52, 0.70) *** | 0.76 (0.64, 0.91) ** | 0.82 (0.61, 1.10) |
| 6-7 | 0.45 (0.41, 0.48) *** | 0.52 (0.43, 0.63) *** | 0.45 (0.27, 0.73) ** | 0.68 (0.32, 1.45) |
| ***P* _for interaction_** | <0.001 | <0.001 | 0.002 | 0.026 |

Adjusted for age, sex, ethnicity, education, Townsend deprivation index (UK biobank), poverty income ratio (NHANES), body mass index, history of cancer, and cardiovascular-kidney-metabolic stage. * *P* < 0.05, ** *P* < 0.01, *** *P* < 0.001

**eTable 6** Summary of sensitivity analysis

|  | **Adjusted hazard ratio (95% confidence interval)** | |
| --- | --- | --- |
|  | **All-cause mortality** | **Cardiovascular mortality** |
| **UK Biobank** | | |
| **Sensitivity analysis 1:** Excluding participants whose outcomes occurred within 2 years after baseline (n=305,444) | | |
| Lifestyle score |  |  |
| 0-2 | 1.00 (Ref) | 1.00 (Ref) |
| 3-5 | 0.51 (0.49, 0.54) *** | 0.49 (0.45, 0.54) *** |
| 6-7 | 0.40 (0.38, 0.42) *** | 0.43 (0.38, 0.48) *** |
| **Sensitivity analysis 2:** Using multiple imputation method to deal with missing covariates (n=310,961) | | |
| Lifestyle score |  |  |
| 0-2 | 1.00 (Ref) | 1.00 (Ref) |
| 3-5 | 0.51 (0.49, 0.53) *** | 0.49 (0.45, 0.54) *** |
| 6-7 | 0.39 (0.37, 0.42) *** | 0.42 (0.38, 0.47) *** |
| **Sensitivity analysis 3:** Excluding participants with a history of cancer at baseline (n=283,691) ^a^ | | |
| Lifestyle score |  |  |
| 0-2 | 1.00 (Ref) | 1.00 (Ref) |
| 3-5 | 0.50 (0.48, 0.52) *** | 0.51 (0.46, 0.56) *** |
| 6-7 | 0.38 (0.36, 0.40) *** | 0.43 (0.38, 0.48) *** |
| **Sensitivity analysis 4:** Using weighted lifestyle score (n=306,831) | | |
| Lifestyle score |  |  |
| Tercile 1 | 1.00 (Ref) | 1.00 (Ref) |
| Tercile 2 | 0.68 (0.66, 0.70) *** | 0.59 (0.55, 0.64) *** |
| Tercile 3 | 0.61 (0.59, 0.64) *** | 0.56 (0.52, 0.61) *** |
| **Sensitivity analysis 5:** Including body mass index in the lifestyle score (n=306,831) ^b^ | | |
| Lifestyle score |  |  |
| 0-2 | 1.00 (Ref) | 1.00 (Ref) |
| 3-5 | 0.54 (0.52, 0.57) *** | 0.50 (0.45, 0.55) *** |
| 6-8 | 0.40 (0.38, 0.42) *** | 0.38 (0.34, 0.43) *** |
| **Sensitivity analysis 6:** Additionally adjusting for medication use (for cholesterol, blood pressure or diabetes) (n=306,831) | | |
| Lifestyle score |  |  |
| 0-2 | 1.00 (Ref) | 1.00 (Ref) |
| 3-5 | 0.51 (0.49, 0.53) *** | 0.49 (0.45, 0.54) *** |
| 6-7 | 0.39 (0.37, 0.41) *** | 0.42 (0.38, 0.48) *** |
|  |  |  |
| **NHANES 2007-2018** | | |
| **Sensitivity analysis 1:** Excluding participants whose outcomes occurred within 2 years after baseline (n=9,687) | | |
| Lifestyle score |  |  |
| 0-2 | 1.00 (Ref) | 1.00 (Ref) |
| 3-5 | 0.72 (0.61, 0.85) *** | 0.75 (0.57, 1.00) |
| 6-7 | 0.45 (0.28, 0.73) ** | 0.56 (0.25, 1.24) |
| **Sensitivity analysis 2:** Using multiple imputation method to deal with missing covariates (n=10,646) | | |
| Lifestyle score |  |  |
| 0-2 | 1.00 (Ref) | 1.00 (Ref) |
| 3-5 | 0.72 (0.62, 0.84) *** | 0.77 (0.59, 1.00) |
| 6-7 | 0.40 (0.25, 0.62) *** | 0.62 (0.31, 1.26) |
| **Sensitivity analysis 3:** Excluding participants with a history of cancer at baseline (n=8,780) ^a^ | | |
| Lifestyle score |  |  |
| 0-2 | 1.00 (Ref) | 1.00 (Ref) |
| 3-5 | 0.66 (0.56, 0.79) *** | 0.64 (0.48, 0.87) ** |
| 6-7 | 0.44 (0.27, 0.72) ** | 0.55 (0.25, 1.23) |
| **Sensitivity analysis 4:** Using weighted lifestyle score (n=9,823) | | |
| Lifestyle score |  |  |
| Tercile 1 | 1.00 (Ref) | 1.00 (Ref) |
| Tercile 2 | 0.68 (0.58, 0.80) *** | 0.78 (0.59, 1.03) |
| Tercile 3 | 0.57 (0.47, 0.69) *** | 0.65 (0.47, 0.90) ** |
| **Sensitivity analysis 5:** Including body mass index in the lifestyle score (n=9,823) ^b^ | | |
| Lifestyle score |  |  |
| 0-2 | 1.00 (Ref) | 1.00 (Ref) |
| 3-5 | 0.74 (0.63, 0.86) *** | 0.75 (0.57, 0.99) * |
| 6-8 | 0.60 (0.44, 0.81) ** | 0.67 (0.39, 1.14) |
| **Sensitivity analysis 6:** Additionally adjusting for medication use (for cholesterol, blood pressure or diabetes) (n=9,821) | | |
| Lifestyle score |  |  |
| 0-2 | 1.00 (Ref) | 1.00 (Ref) |
| 3-5 | 0.72 (0.62, 0.84) *** | 0.74 (0.57, 0.97) * |
| 6-7 | 0.40 (0.25, 0.64) *** | 0.59 (0.28, 1.24) |

Adjusted for age, sex, ethnicity, education, Townsend deprivation index (UK biobank), poverty income ratio (NHANES), body mass index, history of cancer, and cardiovascular-kidney-metabolic stage

^a^ Adjusted for age, sex, ethnicity, education, Townsend deprivation index (UK biobank), poverty income ratio (NHANES), body mass index, and cardiovascular-kidney-metabolic stage

^b^ Adjusted for age, sex, ethnicity, education, Townsend deprivation index (UK biobank), poverty income ratio (NHANES), history of cancer, and cardiovascular-kidney-metabolic stage

* *P* < 0.05, ** *P* < 0.01, *** *P* < 0.001

**eFigure 1** Study flow chart

**eFigure 2** Distribution of healthy lifestyle in the study population
